# The Risk of Epithelial Ovarian Cancer of Women With Endometriosis May be Varied Greatly if Diagnostic Criteria Are Different: A Nationwide Population-Based Cohort Study

**DOI:** 10.1097/MD.0000000000001633

**Published:** 2015-10-02

**Authors:** Wen-Ling Lee, Wen-Hsun Chang, Kuan-Chin Wang, Chao-Yu Guo, Yiing-Jeng Chou, Nicole Huang, Hsin-Yi Huang, Ming-Shyen Yen, Peng-Hui Wang

**Affiliations:** From the Department of Nursing, Oriental Institute of Technology, New Taipei City (W-LL, K-CW); Department of Medicine, Cheng-Hsin General Hospital (W-LL); Department of Obstetrics and Gynecology (W-LL, M-SY, P-HW); Department of Nursing (W-LL, W-HC, P-HW); Institute of Hospital and Health Care Administration and Institute of Public Health, National Yang-Ming University, Taipei, Taiwan (C-YG, Y-JC, NH); Department of Nursing (W-HC); Department of Obstetrics and Gynecology (W-HC, M-SY, P-HW); Biostatics Task Force, Taipei Veterans General Hospital, Taipei, Taiwan (H-YH); Department of Medical Research (P-HW); and China Medical University Hospital, Taichung, Taiwan.

## Abstract

This article aims to test the hypothesis that the risk of epithelial ovarian cancer (EOC) in women with endometriosis might be changed by enrolling different population.

A nationwide 14-year historic cohort study using the National Health Insurance Research Database (NHIRD) of Taiwan and the Registry for Catastrophic Illness Patients was conducted. A total of 239,385 women aged between 20 and 51 years, with at least 1 gynecologic visit after 2000, were analyzed. Cases included women with a diagnosed endometriosis, which was established along a spectrum from at least 1 medical record of endometriosis (recalled endometriosis) to tissue-proved ovarian endometriosis (n = X). Controls included women without any diagnosis of endometriosis (n = 239,385 – X). We used Cox regression, and computed hazard ratios (HRs) with 95% confidence intervals (95% CI) to determine the risk of EOC in patients.

The EOC incidence rates (IRs, per 10,000 person-years) of women with endometriosis ranged from 1.90 in women with recalled endometriosis to 18.70 in women with tissue-proved ovarian endometrioma, compared with those women without any diagnosis of endometriosis (0.77–0.89), contributing to crude HRs ranging from 2.59 (95% CI, 2.09–3.21; *P* < 0.001) to 24.04 (95% CI, 17.48–33.05; *P* < 0.001). After adjustment for pelvic inflammatory disease, infertility, Charlson co-morbidity index, and age, adjusted HRs were ranged from the lowest of 1.90 (95% CI, 1.51–2.37; *P* < 0.001) in recalled endometriosis to the highest of 18.57 (95% CI, 13.37–25.79; *P* < 0.001) in tissue-proved ovarian endometrioma, which was inversely related to the prevalence rate of endometriosis (from the highest of 30.80% in recalled endometriosis to the lowest of 1.54% in tissue-proved ovarian endometrioma).

The risk of EOC in women with endometriosis varied greatly by different criteria used. Women with endometriosis might have a more apparently higher risk than those reported by systematic review and meta-analysis.

## Abstract 2

Supplemental Digital Content is available in the text

## INTRODUCTION

**FIGURE 1:**
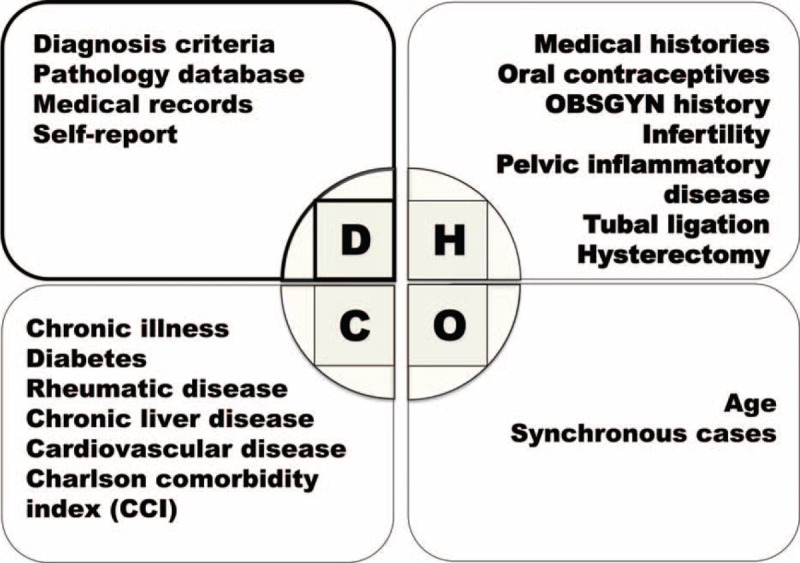
Confounding factors might influence the risk estimation of epithelial ovarian cancer in women with endometriosis.

Dr. Sampson in 1925 proposed a possible correlation between endometriosis and malignant transformation (occurrence of epithelial ovarian cancer [EOC]); thereafter, many epidemiologic studies, including recent systematic reviews^1–9^ and meta-analyses,^3,4,10–12^ indicated that women with endometriosis might have an increased risk of EOC. Although epidemiologic studies have not always supported a positive correlation between endometriosis and EOC,^13–15^ other studies have reported an unusually high risk of EOC in women with diagnosed endometriosis.^16,17^ The question is, why has the risk of EOC in women with endometriosis varied in different studies, ranging from no correlation in Olson study^13^ to the highest hazard ratio (HR) of 12.4 in Buis study.^17^ Our previous study showed that women with a tissue-proved endometriosis had a higher risk of EOC than those without (adjusted HR, 5.62; 95% confidence interval [CI], 3.46–9.14),^18^ which was significantly >1.80 reported by a recent meta-analysis.^11^ Although many hypotheses have been put forth to explain this observation, including the presence of many confounding factors (Fig. 1),^18–25^ one of the most likely reasons might involve the selected population for study. Dr. Buis found that the risk of EOC-associated endometriosis was much higher, if the data of the diagnosed endometriosis were validated by the nationwide pathology database.^17^ The authors suggested that the low-risk estimates of EOC in women with endometriosis from previous studies might be secondary to nondifferential misclassification bias.^17^ To test the hypothesis that different classification of endometriosis might directly contribute to the estimation of the prevalence of endometriosis and a subsequent risk of EOC, we conducted a nationwide historic cohort study of women between 1996 and 2010. For this analysis, we selected all cohorts of women with any diagnosis of endometriosis (ranging from at least 1 medical record of endometriosis to tissue-proved ovarian endometrioma; n = X) were compared with women without any diagnosis of endometriosis (n = 239,385-X) to test the impact of diagnostic criteria of endometriosis on the risk calculation of EOC.

## METHODS

The study population in this retrospective cohort study contained nearly the entire population of Taiwan (23 million inhabitants). The data in the current study were that of the research database of Taiwan's National Health Insurance (NHI) program from 1996 to 2010, which contains 1 million randomly sampled beneficiaries (The Longitudinal Health Insurance Database 2000 [LHID 2000]), and the data in the LHID 2000 are representative of all beneficiaries with regard to age, sex, and insurance cost.^25–27^

This study projected was approved by the Institutional Review Board of Taipei Veterans General Hospital (VGHIRB Number: 2012–12–012BC). We obtained the permission to use the data in the NHI Research Database (NHIRD) from the National Health Research Institute (NHRI) in Taiwan and performed this study. The final target subjects were 239,385 women aged between 20 and 51 years, with at least 1 gynecologic visit after 2000 for analysis. Because no personal identification could be obtained from the data from the NHIRD provided by the Bureau of NHI, Department of Health and managed by NHRI, we could not identify who was who in this study. Therefore, written informed consent was not needed.

**FIGURE 2:**
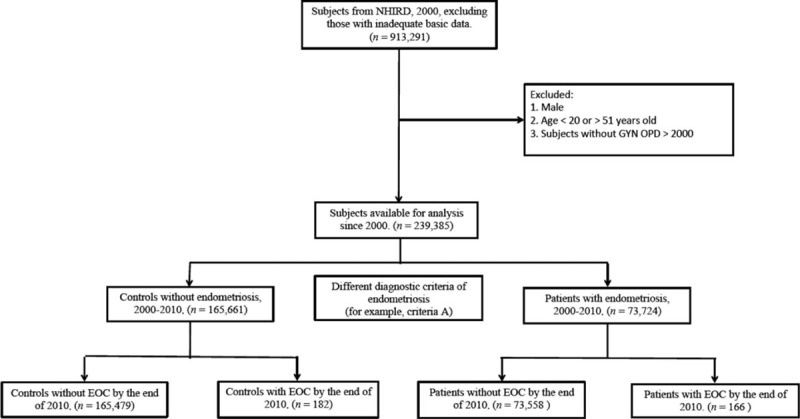
Cohort flow chart illustrating the inclusion and exclusion criteria of participants in the study (Group A: at least one medical record of endometriosis at outpatient clinics or during hospitalization (recalled endometriosis and/or self-reported endometriosis).

The diagnosis of women with endometriosis was based on 13 different criteria (Table 1), ranged from presence of at least 1 medical record of endometriosis (recalled and/or self-reported endometriosis, Figure 2 as an example) to tissue-proved ovarian endometriosis in the administrative dataset, and the remaining women were classified as controls (women without any endometriosis). We identified the women with endometriosis based on medical records using International Classification of Diseases, Ninth Revision, and Clinical Modifications (ICD9-CM) code 617, which was obtained from either inpatient (hospitalization) or outpatient (OPD) systems. Since the patients might have had >1 visit, especially within 1 year, or visited any doctor or been hospitalized, the origin of the medical record of endometriosis (ICD9-CM 617) varied.

**TABLE 1:**
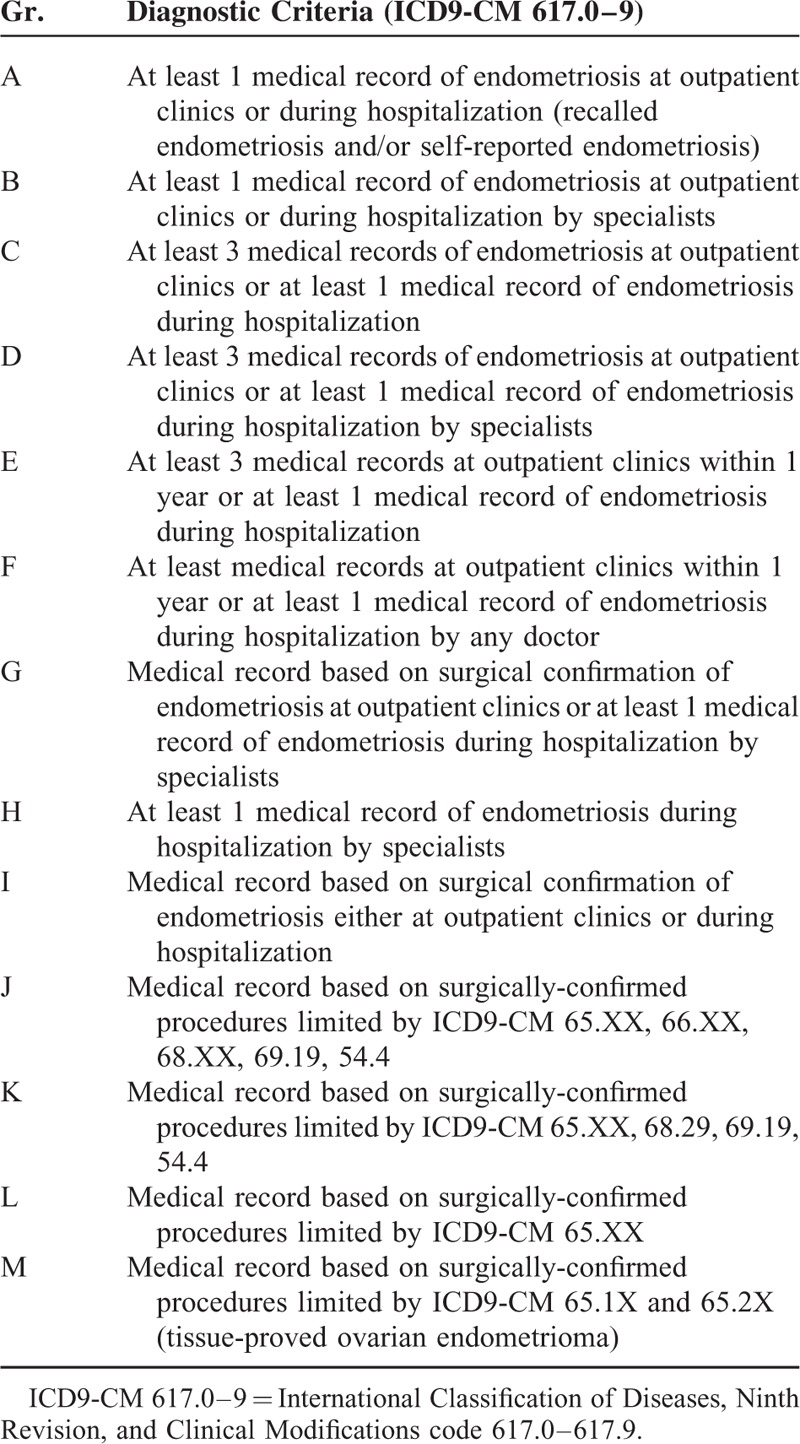
Criteria Used in the Current Study

The following strategy, which has been described before in detail, was used to validate the tissue-proved endometriosis. We recorded surgical treatments for endometriosis, especially limited to the ovary, tube, and peritoneal cavity.^18,25^ Since the surgical interventions, including hysterectomy, bilateral salpingo-oophorectomy, and bilateral oophorectomy contributed to the decreased risk of future EOC, we excluded these subjects, except those women with a diagnosis of invasive EOC during the follow-up period.^18,25^

A total of 13 diagnostic criteria (definition) of endometriosis were used in this study, and all criteria had to fulfill the minimal requirement—having at least 1 medical record of ICD9-CM 617.0–617.9 diagnosis (Table 1 and Fig. 2). The women with endometriosis should be initially detected between 2000 and 2010. That is to say, the index date was the date of the first visit/admission during this period (from 2000 to 2010). For the controls (remains without endometriosis), the index date was the date of the first visit to an obstetric/gynecological provider or admission during the study period.

The diagnosis of EOC was confirmed in inpatients with tissue approval and validated using the major disease files (ICD9-CM 183.0 from the Registry for Catastrophic Illness Patients).^18,28^

### Statistical Analysis

The method for analysis used in the current study has been described before.^18,25^ In brief, starting from the cohort index date, the subjects in the current study were followed until hospitalization with EOC or death, whichever came first, or to the end of the study (December 31, 2010) if no EOC or death had occurred. The censored subjects included patients who did not have a diagnosis of EOC or lost to the follow-up. We used the incidence rate (IR) of EOC to compare women with and without endometriosis, which was tested by the *χ*2 test among subsamples. We used the Cox proportional hazards model to calculate the HR and 95% confidence interval (CI) to determine whether newly diagnosed endometriosis was a risk factor for EOC. The SAS statistical package, version 9.3 (SAS Institute, Cary, NC), and Stata Statistical Software, version 12.0 (Stata Corporation, College Station, TX) were used to perform statistical analyses.

## RESULTS

In all, 348 of the total 239,385 study subjects had EOC between 2001 and 2010. The total person-years of follow-up ranged from 3,228,799 to 3,409,338, based on the different diagnostic criteria (Table 2). The differences in the number of women that obtained a diagnosis of endometriosis, ranging from 73,724 in recalled endometriosis (Group A) to 3782 in tissue-proved ovarian endometrioma (Group M) (Table 2), were due to the different criteria used to diagnose endometriosis. The EOC IR of the women with endometriosis varied greatly, ranging from 1.9 per 10,000 person-years in recalled endometriosis to 18.7 per 10,000 person-years in tissue-proved ovarian endometrioma (Table 2). However, the EOC IR of the women without endometriosis was relatively consistent, ranging from 0.8 to 0.9 per 10,000 person-years and this EOC IR of the women was close to the age-standard IR (age-SIR) of EOC after age adjustment in Taiwan (0.8–0.9 per 10,000 person-years).^28^

**TABLE 2:**
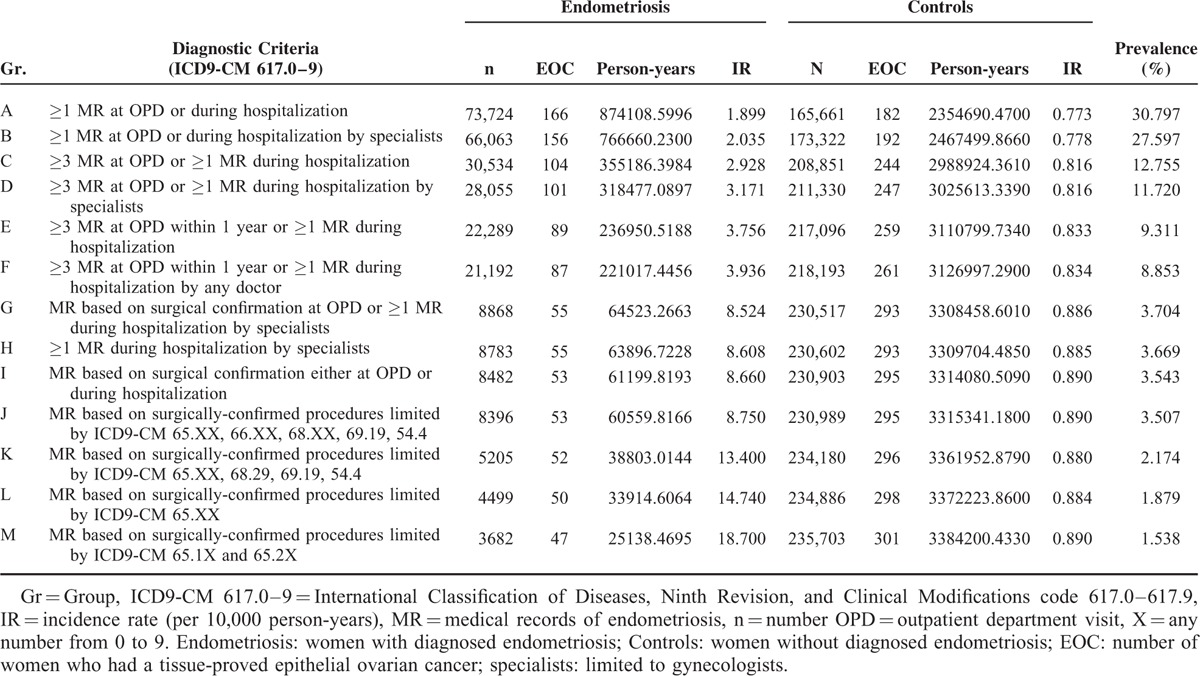
Incidence Rate of Women With and Without Endometriosis, Based on Different Diagnostic Criteria, and Prevalence of Endometriosis in the Current Study Population

Different IRs of EOC in women with endometriosis contributed to the different risk estimation of EOC, with crude HRs ranging from 2.59 (95% CI 2.09–3.21, *P* < 0.001) in recalled endometriosis to 24.04 (95% CI, 17.48–33.05; *P* < 0.001) in tissue-proved ovarian endometrioma. After adjustment of confounders, including pelvic inflammatory disease (PID), infertility and Charlson co-morbidity index (CCI), women with endometriosis still had a higher risk of EOC than women without, with adjusted HRs ranging from 2.83 (95% CI, 2.27–3.52; *P* < 0.001) and 1.81 (95% CI, 1.45–2.25; *P* < 00.001) in recalled endometriosis to 26.39 (95% CI, 19.08–36.49; *P* < 0.001) and 16.46 (95% CI, 11.94–22.71; *P* < 0.001) in tissue-proved ovarian endometrioma (Table 3).

**TABLE 3:**
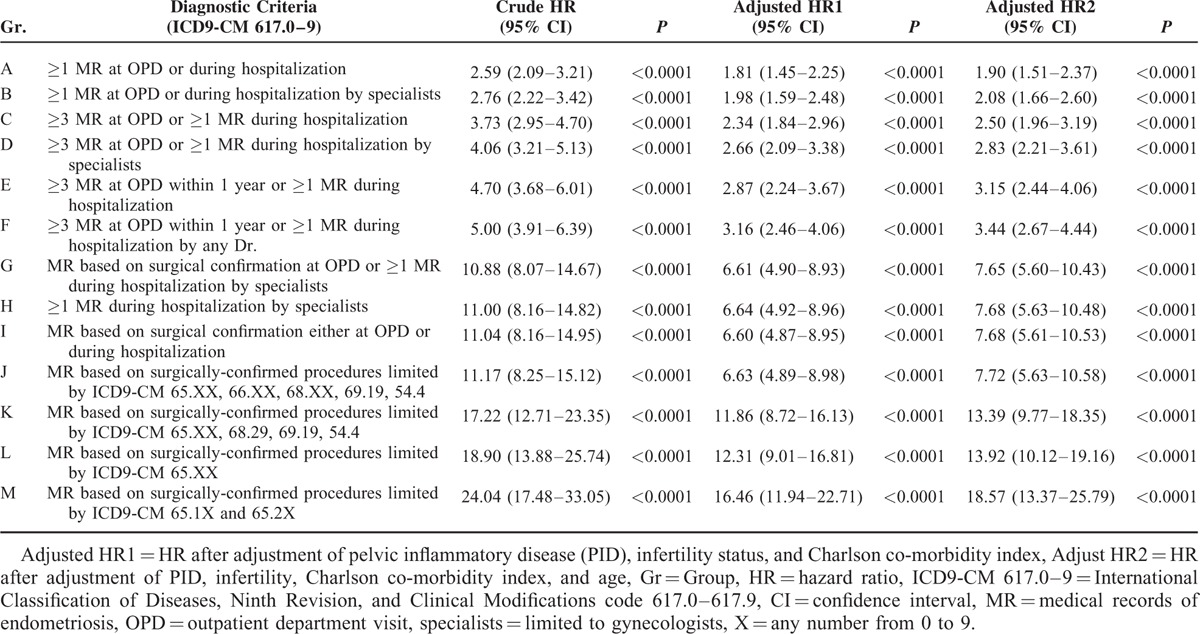
The Risks of Epithelial Ovarian Cancer in Women With Endometriosis, Based on Different Diagnostic Criteria

**FIGURE 3:**
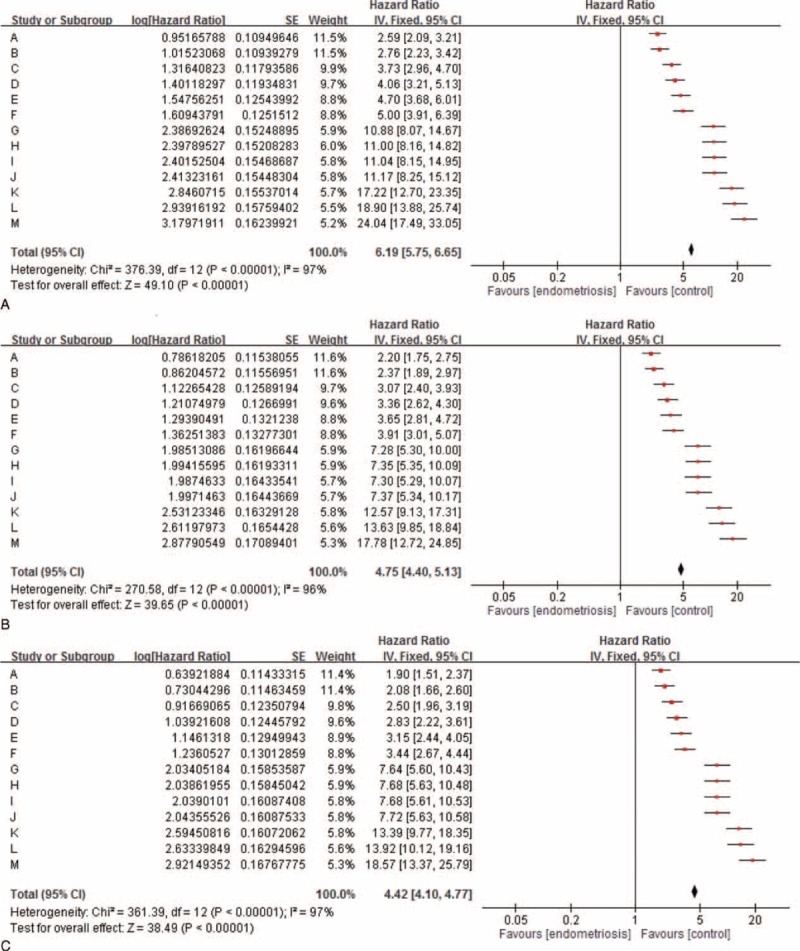
Forest plot assessing overall women with endometriosis, based on 13 different diagnostic criteria in this study and the risk of epithelial ovarian cancer.

Since median age of the women at diagnosis of endometriosis (34–41 vs. 29–30 years; P < .001) and at the subsequent diagnosis of EOC (39–44 vs. 37 years; P < .001) was significantly higher than that of the women without a diagnosis (Supplementary Table 4, http://links.lww.com/MD/A429, which illustrates median age of women with and without endometriosis, based on different diagnostic criteria), we adjusted age for the risk analysis. We then found that women with endometriosis had a constantly higher risk of EOC than women without (adjusted HRs of 1.90 [95% CI, 1.51–2.37; *P* < 0.001) in recalled endometriosis to 18.57 [95% CI, 13.37–25.79; *P* < 0.001] in tissue-proved ovarian endometrioma; Table 3 and Fig. 3).

To investigate the role of follow-up time between enrollment and the occurrence of EOC (interval), we found that the median interval between the cohort index date and the date of EOC for the women with endometriosis varied greatly, ranged from 1203.5 days in recalled endometriosis to 14 days in tissue-proved ovarian endometrioma, but the interval of women without endometriosis was relatively constant, ranged from 3381 to 3469 days (Supplementary Table 5, http://links.lww.com/MD/A429, which illustrates the median interval between enrollment in the cohort and diagnosis of invasive EOC, based on different diagnostic criteria). The difference between the 2 groups was statistically significant (*P* < 0.001). Results suggested that women with endometriosis had a significantly shorter interval in which to develop EOC, compared with the longer interval of women without endometriosis. Since the highest risk of EOC could be identified in the first-year follow-up, the question that the risk of EOC in women with endometriosis might be biased by surveillance was raised. Therefore, we used the Nelson-Aalen cumulative hazard estimates model to evaluate the relationship between enrollment in this cohort and the occurrence of EOC to minimize the bias secondary to surveillance. We found that women with endometriosis had a consistently and persistently higher risk of EOC than women without endometriosis did, regardless of what criteria were used (Fig. 4  ).

**FIGURE 4:**
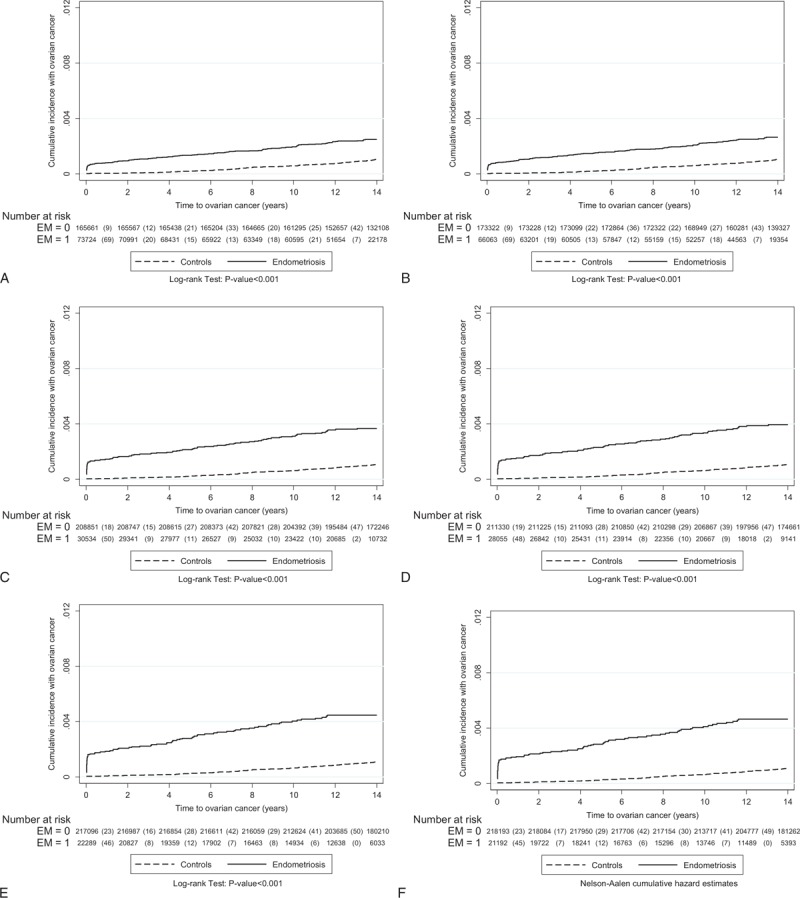
The relationship between enrollment in this cohort and the occurrence of epithelial ovarian cancer was evaluated using the Nelson-Aalen cumulative hazard estimates model. The data are based on the different diagnostic criteria of endometriosis (from Group A: at least one medical record of endometriosis at outpatient clinics or during hospitalization—recalled or self-reported endometriosis to Group M: tissue-proved ovarian endometrioma). The detailed information is shown in the Table 1.

**FIGURE 4 (Continued):**
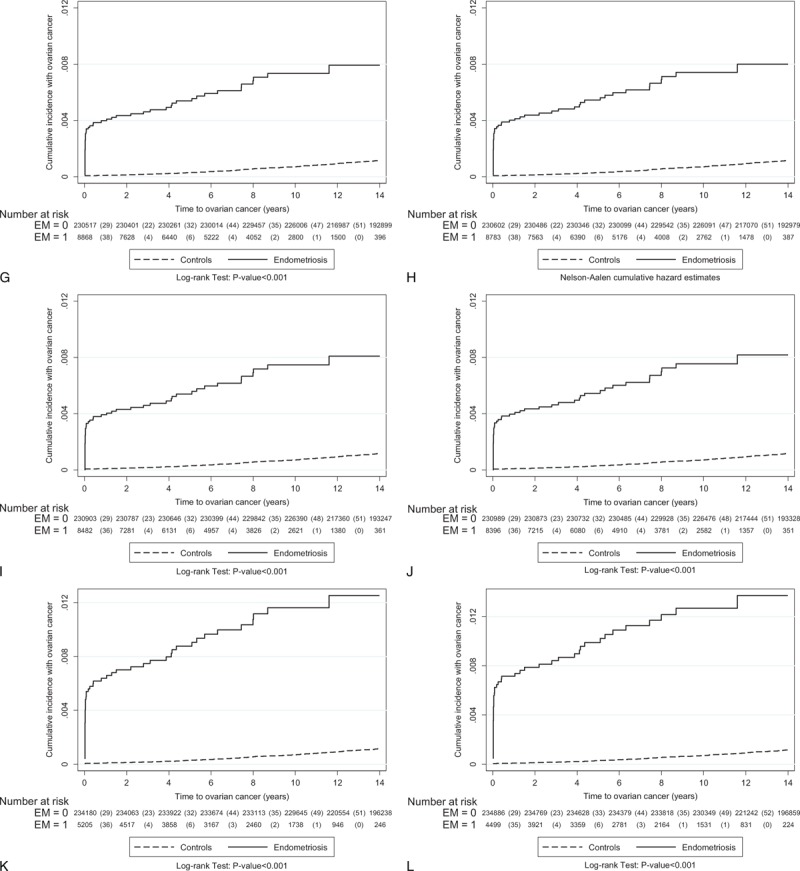
The relationship between enrollment in this cohort and the occurrence of epithelial ovarian cancer was evaluated using the Nelson-Aalen cumulative hazard estimates model. The data are based on the different diagnostic criteria of endometriosis (from Group A: at least one medical record of endometriosis at outpatient clinics or during hospitalization—recalled or self-reported endometriosis to Group M: tissue-proved ovarian endometrioma). The detailed information is shown in the Table 1.

**FIGURE 4 (Continued):**
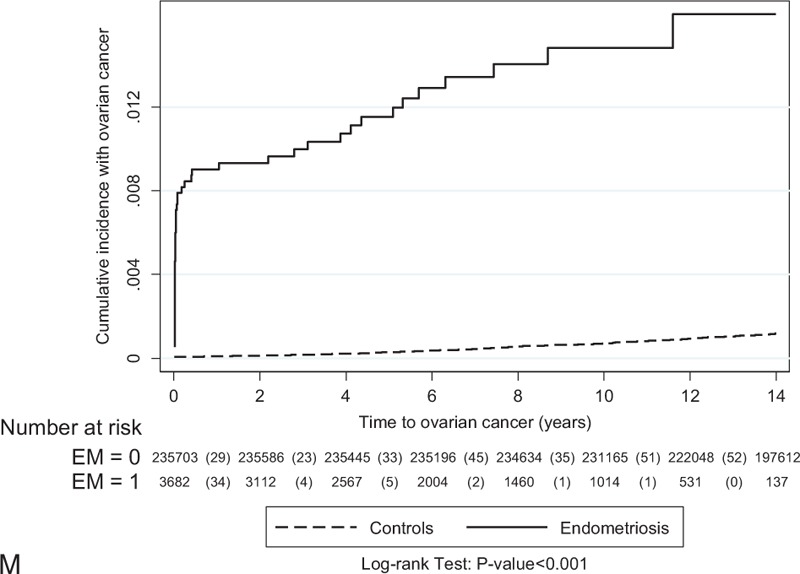
The relationship between enrollment in this cohort and the occurrence of epithelial ovarian cancer was evaluated using the Nelson-Aalen cumulative hazard estimates model. The data are based on the different diagnostic criteria of endometriosis (from Group A: at least one medical record of endometriosis at outpatient clinics or during hospitalization—recalled or self-reported endometriosis to Group M: tissue-proved ovarian endometrioma). The detailed information is shown in the Table 1.

Furthermore, we tested the relationship between the prevalence of women diagnosed with endometriosis and the risk of EOC. Results showed that the prevalence of endometriosis was negatively related to the risk of EOC in women with endometriosis (Table 2). The recalled endometriosis contributed to not only the largest number of patient enrollments (73,724 women) and the highest prevalence of endometriosis (30.797%) in the general population, but also the lowest risk of EOC (IR 1.899). By contrast, the strictest criteria of endometriosis (tissue-proved ovarian endometrioma) not only contributed to the smallest number of patient enrollments (3682 women) and the largest population with the lowest prevalence of endometriosis (1.538%), but also resulted in the highest risk of EOC (IR 18.700) in this population (Supplementary Figure 5, http://links.lww.com/MD/A429, which illustrates a negative correlation between the prevalence of endometriosis the risk of EOC in women with endometriosis, based on different diagnostic criteria).

## DISCUSSION

To date, most observational studies have not considered the use of a reliable diagnostic test to confirm the diagnosis of endometriosis when assessing the risk of development of EOC in women with endometriosis. The diagnosis of endometriosis in systematic reviews and meta-analyses^2–11^ is often based on self-reported endometriosis, which results in a low-risk estimation (HRs ranging from 1.3 to 1.9). The reported strength of association between endometriosis and invasive EOC is well under the level that supports causality, and this weak association may be further reduced after accounting for biases.^29^

Our study showed the dramatic variation of the risk estimation of EOC based on different diagnostic criteria. The largest number of patients enrolling into the endometriosis group contributed to the highest prevalence rate of women with endometriosis and subsequently the lowest risk of EOC estimation in recalled endometriosis. By contrast, the population in ovarian endometrioma excluded those women who might have a pelvic endometriosis, and recalled endometriosis might have enrolled women who might not have endometriosis, but the physicians supposed that these women might have endometriosis. The diagnostic criterion in Group F (at least 3 medical records at outpatient clinics within 1 year or at least 1 medical record of endometriosis during hospitalization by any doctor) might be one of the frequently used criteria to diagnose women with endometriosis. Based on this diagnostic criterion, the prevalence rate was 8.85%, similar to a report stating that women had a lifetime risk ranging from 4% to 9%, or 6% to 15% during reproductive age,^5,30^ and women with endometriosis really had a higher risk of EOC than women without (adjusted HR, 3.44; 95% CI, 2.67–4.44; *P* < 0.001). When tissue-proved endometriosis was used as the diagnostic criterion (eg, Group I: medical record based on surgical confirmation of endometriosis either at outpatient clinics or during hospitalization), the estimated prevalence of endometriosis declined (3.543%), but the risk of EOC was greatly increased, with an adjusted HR of 7.68 (95% CI, 5.63–10.48; *P* < 0.001), compared with the women without endometriosis. Furthermore, if the diagnostic criteria were limited to tissue-proved ovarian endometrioma, the prevalence of ovarian endometrioma was down to a nadir of 1.88% and 1.54%, but the risk of EOC reached the highest levels (HRs, 13.92 and 18.57, respectively). Our findings supported the recent publication from the Netherlands that showed that the risk of EOC associated with endometriosis was much higher in analyses that included information on endometriosis from a nationwide pathology database,^17^ and also supported Kobayashi's findings.^16^ Although Melin et al^19^ found that 1-sided oophorectomy (adjusted OR, 0.19; 95% CI 0.08–0.46), and radical extirpation of all visible endometriosis (adjusted OR 0.30, 95% CI, 0.12–0.74; *P* < 0.001) were protective against the later development of EOC, there is no doubt that women with endometriosis, regardless of the criteria used, indeed had a higher risk of EOC than did women without.

Based on the above findings, risk estimates from most of the previous studies using recalled endometriosis as an inclusion criterion might have a high possibility of misclassification, subsequently contributing to the overestimated prevalence of endometriosis, and the underestimated risk of EOC in women with endometriosis, as concluded by systematic reviews and meta-analyses.^1–12^

The limitation of the study was that we did not evaluate reproductive and hormonal-related factors, such as parity, and the use of combined hormonal contraception or many medical therapies, which are also important to the development of EOC in women. For example, a recent publication by Pearce et al^31^ used control data from 4 US population-based studies to investigate the lifetime risk of EOC after analyzing the joint distribution of risk/protective factor profiles and found that the lifetime risk estimates ranged from 0.35% (95% CI, 0.29–0.42) to 8.78% (95% CI, 7.10–10.9), compared with 1.37% of the general population of US women. There is no doubt that many uncertain confounding factors were not taken into consideration in the current study. Furthermore, the cases of EOC might be misclassified into the “endometriosis” or none-endometriosis groups. However, the age-adjusted IR of EOC in the control group remained constant (0.8–0.9 per 10,000 population). In addition, the age-adjusted IR of EOC in the controls was very similar to age-SIR in the general population in Taiwan,^28^ and also consistent with the reports from the world.^32^ Based on relatively constant age-adjusted IR of EOC in the control group, the calculation of HR in the current study is acceptable. Second, the basic characteristics of the patients were different. For example, the women with endometriosis were significantly older than the women without. After adjustment for age, infertility, PID, and CCI, women with endometriosis still had a higher risk of EOC than women without, regardless of the criteria used.

In conclusion, results of this study, using different strategies to compare the risk of EOC in women with and without endometriosis, reconfirmed a previously reported association between endometriosis and increased risk of EOC, and that the risk of EOC in women with endometriosis might be more apparent. With the use of much stricter criteria for endometriosis (limited to tissue-proved ovarian endometrioma), the prevalence of endometriosis was much lower (1.54%), but the risk of EOC in endometriosis was much higher (IR 18.700). The EOC risk in women with endometriosis (IR ranged from the lowest of 1.899 to the highest of 18.700) was meaningfully negatively correlated with prevalence rate of endometriosis (prevalence rate from the highest of 30.80% to the lowest of 1.54%) in the study population. The findings provided a good explanation for the only 2-fold increase in EOC in systematic reviews and meta-analyses because these data are often based on recalled endometriosis, which results in a low-risk estimation.

## References

[1] Nezhat FR Apostol R Nezhat C New insights in the pathophysiology of ovarian cancer and implications for screening and prevention. *Am J Obstet Gynecol* 2015; pii: S0002-9378 00325-7. 10.1016/j.ajog.2015.03.044 25818671

[2] Kvaskoff M Mu F Terry KL Endometriosis: a high-risk population for major chronic diseases? *Hum Reprod Update* 2015; 21:500–516. 25765863 10.1093/humupd/dmv013 PMC4463000

[3] Zafrakas M Grimbizis G Timologou A Endometriosis and ovarian cancer risk: a systematic review of epidemiological studies. *Front Surg* 2014; 1:14. 25593938 10.3389/fsurg.2014.00014 PMC4286968

[4] Heidemann LN Hartwell D Heidemann CH The relation between endometriosis and ovarian cancer - a review. *Acta Obstet Gynecol Scand* 2014; 93:20–31. 24011403 10.1111/aogs.12255

[5] Vercellini P Viganò P Somigliana E Endometriosis: pathogenesis and treatment. *Nat Rev Endocrinol* 2014; 10:261–275. 24366116 10.1038/nrendo.2013.255

[6] Sayasneh A Tsivos D Crawford R Endometriosis and ovarian cancer: a systematic review. *ISRN Obstet Gynecol* 2011; 2011:140310. 21789283 10.5402/2011/140310 PMC3140029

[7] Wei JJ William J Bulun S Endometriosis and ovarian cancer: a review of clinical, pathologic, and molecular aspects. *Int J Gynecol Pathol* 2011; 30:553–568. 21979592 10.1097/PGP.0b013e31821f4b85 PMC4130217

[8] Munksgaard PS Blaakaer J The association between endometriosis and gynecological cancers and breast cancer: a review of epidemiological data. *Gynecol Oncol* 2011; 123:157–163. 21742370 10.1016/j.ygyno.2011.06.017

[9] Kobayashi H Ovarian cancer in endometriosis: epidemiology, natural history, and clinical diagnosis. *Int J Clin Oncol* 2009; 14:378–382. 19856043 10.1007/s10147-009-0931-2

[10] Somigliana E Vigano’ P Parazzini F Association between endometriosis and cancer: a comprehensive review and a critical analysis of clinical and epidemiological evidence. *Gynecol Oncol* 2006; 101:331–341. 16473398 10.1016/j.ygyno.2005.11.033

[11] Kim HS Kim TH Chung HH Risk and prognosis of ovarian cancer in women with endometriosis: a meta-analysis. *Br J Cancer* 2014; 110:1878–1890. 24518590 10.1038/bjc.2014.29 PMC3974076

[12] Pearce CL Templeman C Rossing MA Webb PM; Ovarian Cancer Association Consortium. Association between endometriosis and risk of histological subtypes of ovarian cancer: a pooled analysis of case-control studies. *Lancet Oncol* 2012; 13:385–394. 22361336 10.1016/S1470-2045(11)70404-1 PMC3664011

[13] Olson JE Cerhan JR Janney CA Postmenopausal cancer risk after self-reported endometriosis diagnosis in the Iowa Women's Health Study. *Cancer* 2002; 94:1612–1618. 11920519 10.1002/cncr.10370

[14] Venn A Watson L Lumley J Breast and ovarian cancer incidence after infertility and in vitro fertilisation. *Lancet* 1995; 346:995–1000. 7475593 10.1016/s0140-6736(95)91687-3

[15] Rossing MA Daling JR Weiss NS Ovarian tumors in a cohort of infertile women. *N Engl J Med* 1994; 331:771–776. 8065405 10.1056/NEJM199409223311204

[16] Kobayashi H Sumimoto K Moniwa N Risk of developing ovarian cancer among women with ovarian endometrioma: a cohort study in Shizuoka, Japan. *Int J Gynecol Cancer* 2007; 17:37–43. 17291229 10.1111/j.1525-1438.2006.00754.x

[17] Buis CC van Leeuwen FE Mooij TM OMEGA Project Group. Increased risk for ovarian cancer and borderline ovarian tumours in subfertile women with endometriosis. *Hum Reprod* 2013; 28:3358–3369. 24014607 10.1093/humrep/det340

[18] Wang KC Chang WH Lee WL An increased risk of epithelial ovarian cancer in Taiwanese women with a new surgio-pathological diagnosis of endometriosis. *BMC Cancer* 2014; 14:831. 25403543 10.1186/1471-2407-14-831 PMC4240825

[19] Melin AS Lundholm C Malki N Hormonal and surgical treatments for endometriosis and risk of epithelial ovarian cancer. *Acta Obstet Gynecol Scand* 2013; 92:546–554. 23560387 10.1111/aogs.12123

[20] Merritt MA De Pari M Vitonis AF Reproductive characteristics in relation to ovarian cancer risk by histologic pathways. *Hum Reprod* 2013; 28:1406–1417. 23315066 10.1093/humrep/des466 PMC3627336

[21] Stewart LM Holman CD Aboagye-Sarfo P In vitro fertilization, endometriosis, nulliparity and ovarian cancer risk. *Gynecol Oncol* 2013; 128:260–264. 23116937 10.1016/j.ygyno.2012.10.023

[22] Vlahos NF Economopoulos KP Fotiou S Endometriosis, in vitro fertilisation and the risk of gynaecological malignancies, including ovarian and breast cancer. *Best Pract Res Clin Obstet Gynaecol* 2010; 24:39–50. 19733123 10.1016/j.bpobgyn.2009.08.004

[23] Modugno F Ness RB Allen GO Oral contraceptive use, reproductive history, and risk of epithelial ovarian cancer in women with and without endometriosis. *Am J Obstet Gynecol* 2004; 191:733–740. 15467532 10.1016/j.ajog.2004.03.035

[24] Brinton LA Lamb EJ Moghissi KS Ovarian cancer risk associated with varying causes of infertility. *Fertil Steril* 2004; 82:405–414. 15302291 10.1016/j.fertnstert.2004.02.109

[25] Chang WH Wang KC Lee WL Endometriosis and the subsequent risk of epithelial ovarian cancer. *Taiwan J Obstet Gynecol* 2014; 53:530–535. 25510696 10.1016/j.tjog.2014.04.025

[26] Shih CJ Wu YL Lo YH Association of hypoglycemia with incident chronic kidney disease in patients with type 2 diabetes: a nationwide population-based study. *Medicine (Baltimore)* 2015; 94:e771. 25906112 10.1097/MD.0000000000000771 PMC4602688

[27] Shih CJ Chen YT Ou SM Urinary calculi and risk of cancer: a nationwide population-based study. *Medicine (Baltimore)* 2014; 93:e342. 25546684 10.1097/MD.0000000000000342 PMC4602593

[28] Chiang YC Chen CA Chiang CJ Trends in incidence and survival outcome of epithelial ovarian cancer: 30-year national population-based registry in Taiwan. *J Gynecol Oncol* 2013; 24:342–351. 24167670 10.3802/jgo.2013.24.4.342 PMC3805915

[29] Vigano P Somigliana E Parazzini F Bias versus causality: interpreting recent evidence of association between endometriosis and ovarian cancer. *Fertil Steril* 2007; 88:588–593. 17320873 10.1016/j.fertnstert.2006.11.180

[30] Tsui KH Lee WL Chen CY Medical treatment for adenomyosis and/or adenomyoma. *Taiwan J Obstet Gynecol* 2014; 53:459–465. 25510683 10.1016/j.tjog.2014.04.024

[31] Pearce CL Stram DO Ness RB Population distribution of lifetime risk of ovarian cancer in the United States. *Cancer Epidemiol Biomarkers Prev* 2015; 24:671–676. 25623732 10.1158/1055-9965.EPI-14-1128 PMC4892114

[32] Jayson GC Kohn EC Kitchener HC Ovarian cancer. *Lancet* 2014; 384:1376–1388. 24767708 10.1016/S0140-6736(13)62146-7

